# Effect of Omega-3 Fatty Acid Supplementation on Broilers’ Health and Meat Quality—Systematic Review

**DOI:** 10.3390/ani16050846

**Published:** 2026-03-08

**Authors:** Peter Ayodeji Idowu, Tshilidzi Cynthia Negogogo, Takalani J. Mpofu

**Affiliations:** Department of Animal Sciences, Faculty of Science, Tshwane University of Technology, Private Bag X680, Pretoria 0001, South Africa; ayodejiidowuolu@gmail.com (P.A.I.); negogogotc@gmail.com (T.C.N.)

**Keywords:** broiler nutrition, omega-3 fatty acids, EPA, DHA, meat quality, immune modulation, oxidative stability, systematic review

## Abstract

Omega-3 polyunsaturated fatty acids are increasingly applied as functional nutrients to improve broiler health, metabolic resilience, and meat quality. However, studies remain inconsistent regarding optimal inclusion levels and their efficacy. This systematic review was conducted following PRISMA 2020 guidelines. This study included 69 experimental studies that met predefined eligibility criteria, requiring broiler-specific trials with clear reporting of omega-3 source, inclusion level, and measurable outcomes related to growth, immune or antioxidant function, gut morphology, fatty acid composition, or meat quality. Searches were performed in Scopus and Web of Science for peer-reviewed articles published between 2020 and 2025. Across studies, supplementation with fish oil, flaxseed, or algal oil consistently increased the deposition of long-chain omega-3 fatty acids, eicosapentaenoic acid (EPA) and docosahexaenoic acid (DHA) in broiler tissues. Omega-3 inclusion improved immune modulation, gut morphology, and antioxidant enzyme activity while reducing inflammatory markers and lipid peroxidation. Meat quality benefits included lower n-6:n-3 ratios by improving tenderness and juiciness and enhanced oxidative stability with no detrimental effects on growth performance. Nevertheless, responses varied with broiler strain, dietary formulation, feeding phase, antioxidant protection, and environmental conditions. As high levels of marine oils remained susceptible to oxidative degradation. Overall, omega-3 supplementation represents a viable and scalable precision-nutrition strategy for enriching poultry meat and supporting broiler health. Future research should prioritize cost-effective algal sources, establish phase-specific inclusion thresholds across genetic lines, and evaluate synergy with natural antioxidants and bioactive compounds to optimize oxidative stability, shelf life, and consumer acceptance.

## Simple Summary

This review evaluated the effects of supplementing broiler diets with omega-3 fatty acids sourced from fish oil, flaxseed, and microalgae. Omega-3 inclusion consistently increased the concentration of beneficial long-chain fatty acids in meat and improved immune function, gut health, and antioxidant status in broilers. Positive effects were also observed on meat tenderness, juiciness, and oxidative stability during storage. However, the magnitude of these responses varied with breed, diet formulation, and production conditions. High levels of marine-derived oils may increase lipid oxidation if not supported by adequate antioxidant protection. Overall, omega-3 enrichment represents a viable approach to enhancing broiler health and meat quality. Future work should focus on cost-effective algae-based omega-3 sources and their combination with natural antioxidants to improve meat stability.

## 1. Introduction

Broiler chicken production is one of the most dynamic and economically significant sectors of global animal agriculture [1]. It contributes significantly to food security, nutritional adequacy, and socioeconomic development [1]. Its success is driven by the broiler’s rapid growth rate, efficient feed conversion ratio, short production cycle, and relatively low production cost [2,3,4]. However, the intensive production systems used to support this growth also introduce several challenges. These include increased metabolic stress, oxidative imbalance, and immunosuppression, all of which ultimately compromise broilers’ health, welfare, and carcass quality [3,5]. These physiological challenges create a need for innovative nutritional strategies. Such interventions aim to improve productivity and meat quality while aligning with consumer expectations for healthier, functionally enriched animal products [5]. Among the emerging nutritional strategies, omega-3 fatty acid supplementation has received increasing attention for its multifaceted benefits in broiler production [6,7,8]. Omega-3 fatty acids are a family of essential long-chain polyunsaturated fatty acids (PUFAs). This includes alpha-linolenic acid (ALA; 18:3n-3), primarily found in plant-based sources such as flaxseed and canola. It contains biologically active eicosapentaenoic acid (EPA; 20:5n-3) and docosahexaenoic acid (DHA; 22:6n-3), which are abundant in fish oil and marine algae [5,9,10,11,12,13]. These fatty acids are key components of cell membranes, where they help maintain membrane integrity, modulate inflammation, enhance immune function, and support normal cardiovascular and neural activity [5,10,12]. Omega-3 PUFAs added to poultry diets can improve growth, immunity, gut health, and oxidative balance. They also enrich broiler meat with healthy fatty acids that are beneficial for humans [14,15].

Conventional poultry feeds are high in omega-6 fatty acids from oils like corn and soybean, but they contain very little omega-3 [7]. This creates an unhealthy omega-6:omega-3 ratio that often goes above 15:1. Such an imbalance can increase inflammation and lower the nutritional quality of poultry meat [5,6,10,11,12]. In lieu of this, improving the omega-6:omega-3 balance has become an important goal in broiler nutrition [8,16], especially to ensure a sustainable and cost-effective strategy by enhancing metabolic resilience, improving the lipid composition and oxidative stability of broiler meat [17,18]. Studies have proven that dietary inclusion of flaxseed, fish oil, or microalgae elevates tissue levels of EPA and DHA and modulates the expression of hepatic genes related to lipid metabolism [5,19].

Despite advances in recognizing omega-3 fatty acid metabolism in poultry, variations in bioavailability and oxidative stability remain major challenges. Moreover, the economic feasibility and sensory implications of omega-3-enriched poultry products require further optimization to ensure market acceptance and production sustainability [20]. Therefore, the exploration of optimal sources, supplementation levels, and metabolic pathways of omega-3 fatty acids in broiler chickens is critical for improving health, production efficiency, and meat quality of broiler chickens [21,22,23].

To address these knowledge gaps, this review provides an integrated synthesis of current evidence on omega-3 chemistry, dietary sources, metabolic pathways, and functional effects in broiler nutrition. It further evaluates how omega-3 supplementation influences growth performance, immunity, gut function, and meat quality. This review systematically analyses studies published between 2020 and 2025 to provide an updated assessment of omega-3 nutritional strategies in broiler production. It highlights their role in advancing precision nutrition, developing functional poultry products, and supporting sustainable production systems.

## 2. Materials and Methods

### 2.1. Study Design and Protocol

**Figure 1:**
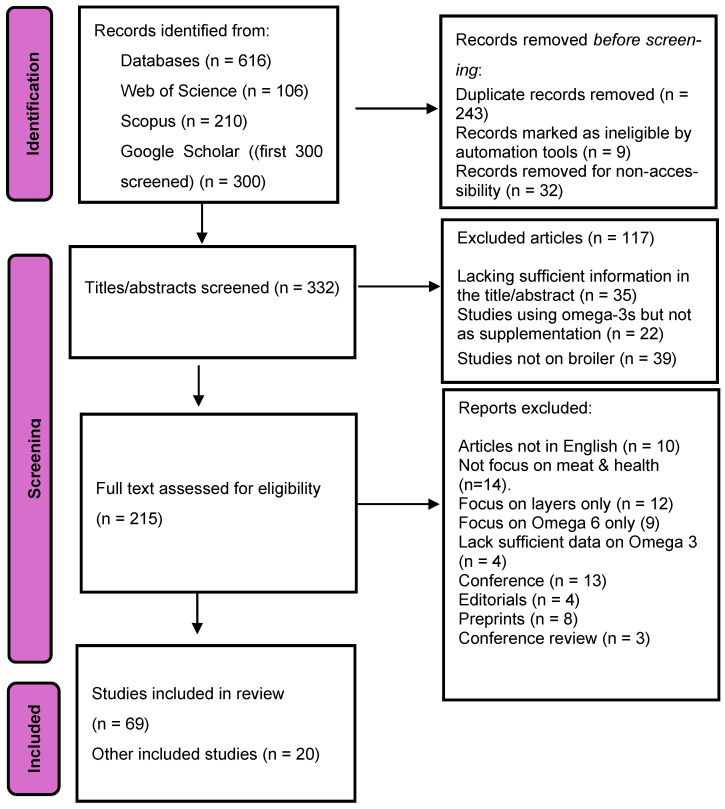
PRISMA flow chart for Omega-3 (2020–2025). Rethlefsen and Page [24].

This systematic review was conducted using the Preferred Reporting Items for Systematic Reviews and Meta-Analyses (PRISMA) 2020 guidelines to ensure methodological transparency and rigor (Figure 1) [24]. The review protocol, which entails objectives, eligibility criteria, and a planned synthesis approach, was developed a priori; however, it was not registered on PROSPERO or any other registry due to the absence of animal-nutrition review categories. The objective of the review was to consolidate and critically evaluate recent evidence (2020–2025) on the chemistry, metabolism, and functional effects of omega-3 fatty acid supplementation in broiler chickens, with emphasis on performance, health indices, and meat quality.

### 2.2. Data Sources and Search Strategy

A comprehensive, multi-database search was conducted in Web of Science (Core Collection), Scopus, and Google Scholar for peer-reviewed studies published between January 2020 and October 2025. Searches used Boolean operators, phrase searching, and database-specific field tags (Table 1). Google Scholar was used as a supplementary source to identify grey-indexed or recently published articles; due to extremely high hit counts, only the first 300 results sorted by relevance were screened using advanced search, following established systematic-review practice [25]. All retrieved records were exported into Mendeley Reference Manager, where automatic and manual duplicate removal was performed.

### 2.3. Eligibility Criteria, Screening, and Data Management

The eligibility criteria for this review were established a priori to ensure consistency and transparency. Studies were included provided they were experimental in vivo trials conducted using broiler chickens (*Gallus gallus domesticus*). To qualify, each study had to clearly describe the omega-3 source, such as fish oil, flaxseed, linseed, chia, algal oil, camelina, or other relevant ingredients. In addition, eligible studies were required to report quantitative outcomes related to growth performance, immune response, antioxidant status, gut morphology, fatty acid composition, or meat quality. Only articles published in English, within peer-reviewed journals, and between the years 2020 and 2025 were considered. Studies were excluded if they involved other poultry species except broilers, including layers, quail, or ducks. Non-experimental papers, such as reviews, editorials, conference abstracts, or preprints, were also excluded, as were studies that focused solely on omega-6 manipulation without assessing omega-3 effects. Research lacking sufficient extractable data on omega-3-related outcomes was not eligible for inclusion. In addition, publications in languages other than English were excluded from the review (Figure 1).

The screening process was conducted in two phases to ensure rigorous evaluation of all identified studies. In Phase 1, two independent reviewers (TJ and PA) screened the titles and abstracts to determine preliminary eligibility. Phase 2 involved a full-text assessment based on the predefined inclusion and exclusion criteria. Any disagreements between the reviewers were resolved through consensus to maintain objectivity. Data from the included studies were then extracted using a standardized Excel sheet, capturing study characteristics, methodological features, and all relevant outcome measures.

### 2.4. Risk of Bias and Quality Assessment

Risk-of-bias assessment was conducted using the SYRCLE RoB tool, version 2 (RoB 2), adapted for animal experiments. Domains assessed included randomization, allocation concealment, blinding, incomplete outcome reporting, selective reporting, and other biases. Each study was categorized as low, unclear, or high risk of bias.

## 3. Results and Discussion

### 3.1. Overview of Literature Search and Study Characteristics

A total of 69 studies met the predefined inclusion criteria and were included in the final synthesis (Figure 1). The initial search identified 616 records, of which 243 duplicates were removed. After screening titles and abstracts, 215 full-text articles were assessed, and 69 were ultimately retained in line with PRISMA 2020 guidelines. The included studies were published articles in research on omega-3 fatty acid supplementation in broiler chickens between January 2020 and October 2025.

Across the eligible studies, the most used broiler strains were Ross 308 (60%), Cobb 500 (25%), and Hubbard or Arbor Acres (10%), with trial durations ranging from 28 to 49 days. The main omega-3 sources reported included flaxseed or linseed oil/meal, fish oil (EPA and DHA), microalgae such as *Chlorella vulgaris*, *Aurantiochytrium limacinum*, and *Schizochytrium* spp., as well as chia seed, purslane meal, camelina, rice bran, various plant-oil blends, and several novel or combined lipid sources such as turmeric with PUFA, Persia Fat, and echium oil. Dietary inclusion levels ranged from 0.05% to 5.0%, equivalent to approximately 500–5000 mg/kg, with supplementation typically applied from the starter to finisher phases (28–42 days).

### 3.2. Sources and Chemistry of Omega-3 Fatty Acids

The physicochemical and biological properties of lipids are fundamentally determined by their fatty acid composition, chain length, and degree of unsaturation [26]. Fatty acids can be broadly categorized as saturated, monounsaturated, or polyunsaturated, depending on the number of double bonds present along their hydrocarbon chain [7]. Saturated fatty acids contain no double bonds, whereas unsaturated fatty acids possess one or more double bonds, which markedly reduce their melting points compared to saturated fatty acids [7,27]. The configuration of these double bonds further affects lipid functionality; trans fatty acids exhibit higher melting points and lower fluidity than their cis isomers due to differences in molecular packing [28].

Omega-3 (n-3) fatty acids represent a class of long-chain PUFAs characterized by the presence of the first double bond between the third and fourth carbon atoms from the methyl (ω) end of the carbon chain [6,7]. Structurally, omega-3 fatty acids contain a terminal carboxylic acid group and a methyl group, with a hydrocarbon chain that varies in length and desaturation [29]. The nutritionally significant members of this family include alpha-linolenic acid (ALA; C18:3n-3), eicosapentaenoic acid (EPA; C20:5n-3), and docosahexaenoic acid (DHA; C22:6n-3) [29]. Variations in their carbon chain length and number of double bonds influence their oxidative stability, membrane fluidity, and metabolic functions in biological systems [30,31].

Omega-3 PUFAs are derived from both terrestrial and aquatic ecosystems (Table 2). Plant-derived sources mainly supply ALA, which serves as the metabolic precursor for long-chain derivatives such as EPA and DHA. Major ALA-rich plant sources include flaxseed (*Linum usitatissimum*), chia (*Salvia hispanica*), perilla (*Perilla frutescens*), hemp (*Cannabis sativa*), and walnut (*Juglans regia*) [30,32,33]. In contrast, marine sources such as fish oil, krill oil, and microalgae, provide EPA and DHA that bypass the rate-limiting enzymatic desaturation and elongation processes [34,35].

Also, stearidonic acid (SDA; C18:4n-3) has gained prominence as a sustainable intermediate omega-3 fatty acid [7,36]. This is due to its ability to efficiently convert to EPA without requiring Δ6-desaturase, the rate-limiting enzyme in ALA metabolism [30,37]. Notable SDA-rich botanical sources include *Echium plantagineum* (12–15% SDA), *Buglossoides arvensis* (up to 20%), *Primula florindae* (11–14%), and *Cannabis sativa* (1–3%) [30]. Among these, *Buglossoides arvensis* (Ahiflower) has emerged as a promising commercial crop due to its balanced n-3/n-6 ratio, high oil yield, and favorable agronomic adaptability [36]. Within marine ecosystems, microalgae such as *Schizochytrium*, *Crypthecodinium cohnii*, and *Phaeodactylum tricornutum* are the primary producers of long-chain omega-3 PUFAs [38,39]. These microalgae form the foundational trophic source of DHA and EPA in aquatic food webs. They are also harnessed for sustainable production in nutraceutical, pharmaceutical, and aquafeed industries [34,35]. Cultivation of microalgae under heterotrophic conditions offers productivity levels of two to three orders of magnitude higher than autotrophic systems. Nevertheless, enhancing cost-effective carbon sources remains a challenge for large-scale production. Table 2 shows detailed examples of most widely used Omega-3 fatty acids in broiler production.

**Table 2:** Sources and Chemistry of Omega-3 Fatty Acids used in Broiler production.

References	Fatty Acid	Chemical Formula	Common Name	Natural Sources	Biological Significance
[27]	(ALA; 18:3n-3)	C_18_H_30_O_2_	Essential omega-3 precursor	Flaxseed, chia, perilla, canola, soybean, walnut	Precursor for long-chain PUFA synthesis
[40]	Stearidonic acid (SDA; 18:4n-3)	C_18_H_28_O_2_	Intermediate omega-3 PUFA	*Echium*, *Buglossoides arvensis*, *Primula*, hemp	Bypasses Δ6-desaturase step and efficient EPA precursor
[41]	Eicosapentaenoic acid (EPA; 20:5n-3)	C_20_H_30_O_2_	Long-chain PUFA	Fish oil, krill oil, microalgae (*Schizochytrium*, *Nannochloropsis*)	Anti-inflammatory and cardiovascular benefits
[42]	Docosapentaenoic acid (DPA; 22:5n-3)	C_22_H_34_O_2_	Intermediate between EPA and DHA	Marine fish, seal oil, microalgae	Modulates inflammation and vascular function
[43]	Docosahexaenoic acid (DHA; 22:6n-3)	C_22_H_32_O_2_	Long-chain PUFA	Fish oil, algal oil, krill	Neural development, membrane integrity and cognitive health

### 3.3. Omega-3 Supplementation Strategies in Broiler Diets

Fish oil is one of the widely used and efficient sources of omega-3 [10,11]. It provides eicosapentaenoic acid (EPA; 20:5n-3) and docosahexaenoic acid (DHA; 22:6n-3), which are readily deposited in the broiler tissues [31,44,45]. Nevertheless, there are growing concerns about its sustainability, oxidative instability, and potential sensory alterations in meat. This has led to the need to explore alternative plant and algal sources [5,37]. Flaxseed and flaxseed oil are rich in alpha-linolenic acid (ALA; 18:3n-3). Also, they represent one of the most common plant-based sources of omega-3s in broiler nutrition. ALA can be metabolically elongated and desaturated to EPA and DHA through the action of Δ6-desaturase and elongase enzymes. Nevertheless, this conversion is generally ineffective in chickens [46,47]. Nonetheless, flaxseed inclusion has been shown to improve hepatic n-3 lipid species and upregulate genes involved in fatty acid oxidation and lipid metabolism, such as FADS1, ELOVL5, and PPARα [44,48]. In contrast, algal oil provides a direct and renewable source of DHA, thereby providing an alternative to fish oil with superior oxidative stability and fewer sensory side effects in meat [49]. The choice of omega-3 source thus depends on the intended outcome. Precisely, flaxseed is adequate for ALA enrichment and endogenous conversion. On the other hand, fish or algal oil is adequate for direct deposition of long-chain PUFAs (EPA and DHA). The dietary inclusion rate of omega-3 fatty acid supplements in broiler feed ranges between 0.5% and 3%, with optimal enrichment generally achieved at 1–2% inclusion during the final 2–4 weeks before slaughter [22]. Inclusion above these levels may negatively affect feed intake and growth performance due to reduced palatability or energy density [50,51].

### 3.4. Growth Performance and Feed Efficiency

Supplementation with 1–3% fish oil in drinking water significantly increased body weight gain (BWG) and improved feed conversion rate (FCR) in Ross 308 broilers during a 30-day feeding period [44]. Similarly, inclusion of salmon-oil concentrate at 0.5% under high-stocking-density stress ameliorated crowding-induced growth. The study further observed enhanced metabolizable energy and nutrient digestibility [28]. In another study, fish oil incorporated at 4% of the diet adjusted the n-6:n-3 ratio to 1.5–4.1. This further improved the final body weight and feed efficiency of the broiler chicken [52]. Collectively, these data indicate that long-chain omega-3 supplementation between 0.5 and 4% of the diet enhances feed efficiency, particularly under physiological or environmental stress. Nevertheless, using Tuna oil to replace rice bran oil with an inclusion level of 1.5%, 3.0%, and 4.5%, respectively, in Korat chicken (broiler) had no significance on growth performance [53]. In broilers fed *Aurantiochytrium limacinum* at 1–2% of the diet, muscle DHA content increased without adverse effects on growth when supplementation was restricted to the finisher phase [54]. However, continuous whole-life inclusion at similar levels slightly reduced feed efficiency. This validates the need for phase-targeted application to optimize performance outcomes. In another study, *Chlorella vulgaris* supplementation up to 1% of the diet had no significant effect on growth rate or feed intake [15]. Diets in broiler reformulated to achieve narrow n-6:n-3 ratios (1.5–4.1) using linseed or blended vegetable oils at approximately 4% inclusion improve BWG and FCR. [55]. Conversely, trials employing flaxseed meal at 4% [33], extruded linseed–pea mixtures at 30% [56] or *sacha inchi* oil at 2% [50] reported no significant influence on growth or feed utilization. These results suggest that ALA sources require either ratio optimization or co-supplementation with long-chain n-3 fatty acids to translate into measurable growth performance.

Replacement of palm oil with rice bran oil at 5% improved feed efficiency and omega-3 deposition in Ross 308 broilers [51]. Furthermore, commercial omega-3 additives of 5–10 kg/feed enhanced BWG and FCR in Hubbard birds [57]. Similarly, combined supplementation of fish, flaxseed, algal, and echium oils at 10% of the diet, maintained growth performance [58]. By contrast, polyphenol-rich additives such as rosemary or turmeric showed no measurable impact on growth rate or feed efficiency [57,59]. Nevertheless, no significant response was observed in studies using flaxseed oil versus fish oil [60], extruded linseed blends [56], microalgal *Chlorella* [15] and botanical co-supplements [58,59]. This response was attributed to low inclusion levels (<0.5%), short supplementation period (<21 days), and/or diets already meeting energy and amino-acid requirements (Table 3). Overall, consistent improvements in growth performance were achieved with dietary long-chain omega-3 fatty acids at inclusion rates between 0.5 and 4% of feed, particularly when environmental stressors were present, or n-6:n-3 ratios were appropriately balanced [22,50,57,61]. These improvements appear to be due to enhanced nutrient digestibility, under both normal and stress conditions [47,51]. In contrast, plant-based ALA sources (flaxseed or linseed) had no direct impact on improving broiler’s growth. Thus, an integrated lipid formulation, such as combining small amounts of preformed EPA/DHA with ALA-rich oils or antioxidants may achieve optimal cost-to-performance balance [35]. Recently, Hassan et al. [62] reported that dietary hemp seed, hemp oil, and cannabinoid-rich fractions improve productive performance across poultry and ruminant species. Future research should refine phase-specific omega-3 dosing strategies to enhance ALA conversion efficiency. Also, systematic comparisons between omega-3 fatty acids and lipophilic immunomodulators such as hemp-derived cannabinoids are needed to optimize next-generation feeding systems aimed at improving broiler growth and feed efficiency.

### 3.5. Immune and Antioxidant Responses

Evidence across broiler studies indicates that dietary omega-3 fatty acids influence immune and antioxidant responses [62,65,67,68,69]. Cobb 500 broilers supplemented with 19 g/kg flaxseed oil or 50 g/kg fish oil had enriched levels of ALA, DPA, and DHA-derived fatty acids in splenic and peripheral blood mononuclear cells [68]. Also, enhanced cytotoxic activity, in the flaxseed-fed group was observed [68]. Similarly, in Ross 308 birds fed 2–4% fish oil or linseed oil, cytokine expression profiles shifted toward a more immunostimulatory profile, with increased IL-1β, IL-6, and IFN-γ. This was achieved when dietary n-6:n-3 ratios declined from approximately 40:1 to 3:1 [70]. These studies suggest that both dosage and fatty-acid ratios shape the immune response to omega-3 supplementation. However, trials using extruded linseed-pea mixtures at 30% inclusion in Cobb or Ross strains (with or without *Lactobacillus acidophilus*) reported no changes in systemic immunity or oxidative biomarkers [56]. This shows that Omega-3 benefits are not universal. Non-PUFA bioactives also contribute significantly to immune modulation. In broilers supplemented with *Artemisia argyi,* a polyphenol and flavonoid-rich botanical plant-derived tannins. An increase in IgG, IgA, IgM, and sIgA with reductions in IL-1β, IL-6, and TNF-α were observed [71]. Also, the study observed an increase in antioxidant enzyme activities with improved immune cells [72]. Broiler receiving yeast-derived β-glucans or mannans, observed consistent improved SCFA production and regulated lung cytokine expression through gut–lung crosstalk [73]. These outcomes support the idea that non-PUFA compounds, even at moderate inclusion rates, can modulate host immunity through antioxidant enhancement and microbiota-immune interactions (Table 4). Omega-3 supplementation also lowers cholesteryl ester transfer protein activity, thereby reducing the transfer of high-density lipoprotein (HDL) cholesteryl esters to apoB-lipoproteins and promoting higher circulating HDL-cholesterol [74]. Omega-3 supplementation increased EPA and DHA incorporation into HDL and altered its lipid and enzyme composition. These changes were accompanied by increases in large HDL particles, reductions in small HDL subfractions, and lower (Cholesteryl Ester Transfer Protein) CETP activity [75]. This shows an improved HDL functional quality. Although the intervention did not enhance HDL antioxidant capacity, the shifts in HDL composition and enzyme activity (PON1 and Apo AI associations) suggest that omega-3 enrichment contributes to favorable remodeling of HDL properties (Table 4). Clinical trials further confirm that omega-3 supplements produce increases in HDL-C [76]. Fish oil–induced improvements in HDL efflux capacity correlate strongly with plasma EPA, providing direct mechanistic evidence linking omega-3 incorporation to enhanced HDL functionality. Transcriptomic analyses further strengthen the mechanistic evidence. In broilers supplemented with omega-3 sources, Wang et al. [10,12] demonstrated post-transcriptional regulation of genes associated with oxidative stress (GPX7, HSPB7), mucosal immunity (MUC2, CXCR1), leukocyte activation, and chemokine signalling. Further study expanded this understanding by identifying lipid-responsive sites within MAPK-, cytokine-, and redox-related networks through integrated multi-omics [77]. Together, these studies confirm that immune and antioxidant modulation occurs through omega fatty acids supplementation and through coordinated regulation of immune gene networks. In Ross 308 broilers supplemented with 4 g/kg turmeric plus omega-3, reductions in TBARS and improved DHA deposition were observed [59]. Likewise, rosemary and blackcurrant extracts, when combined with PUFA diets, reduced MDA levels [58] Algal DHA from *Aurantiochytrium* at 1–2% inclusion maintained stable TBARS and peroxide values during storage [54]. These standardized outcomes illustrate that antioxidant phytochemicals and algal lipids complement omega-3 functionality, especially under conditions of oxidative stress. Collectively, the standardized evidence across strains, inclusion levels, and sample sizes shows that omega-3 PUFA, alternative lipid sources, and plant-derived bioactives improve immune robustness and antioxidant stability through interrelated mechanisms. These include membrane lipid remodeling, modulation of cytokine and inflammatory pathways, enhancement of endogenous antioxidant enzymes, and mitigation of stress-induced immune suppression. Yet, variation in study outcomes underscores that responses depend on the specific lipid source, inclusion rate, fatty-acid composition, and environmental or physiological stressors (Table 4). Future research should integrate transcriptomics and systems-biology approaches for definitive lipid-responsive regulatory networks. Also, to refine precision-feeding strategies that optimize immune and oxidative resilience in broiler production.

### 3.6. Meat Quality and Fatty Acid Composition

Across both Ross 308 and Cobb 500 broilers, fish oil supplementation at 1–4% y increased the proportion of EPA (C20:5n-3) and DHA (C22:6n-3) in breast and thigh muscles [26,51,82]. Similar improvement was observed with *Chlorella vulgaris* (0–1%), with an improved α-linolenic acid retention and enhanced meat color stability [15]. This explains the potential of non-fish sources to improve meat quality. In feeding trials supplemented with fish and rice bran oil, improved juiciness and tenderness, with elevated cooking loss at higher oil inclusion, were observed [51]. In another study, diets enriched with 0.5% salmon oil under high stocking density improved water-holding capacity and decreased MDA concentrations in breast muscle [28]. Both studies recommend that long-chain n-3 PUFA may confer oxidative protection during growth and storage phases.

Turmeric-supplemented diets reduced TBARS values in thigh meat and enhanced colour stability [59,82]. On the other hand, the use of rosemary and blackcurrant extracts effectively minimizes lipid oxidation in PUFA-enriched meats during frozen storage without affecting the meat texture or pH [17]. The use of rosemary or blackcurrant extract with PUFA-enriched diets effectively decreased lipid oxidation during frozen storage without altering texture or pH [17]. Beyond oxidative stability, broader meat quality improvements were observed when probiotics were co-supplemented with an Omega-3 fatty acid (30% extruded linseed-pea) mixture. Lowered atherogenic and thrombogenic indices were observed, which indicates healthier lipid profiles and superior nutritional quality [56]. Replacement of palm oil with rice bran oil of 5%, enhanced the ω-3 content, increased ALA levels, and reduced the n-6:n-3 ratio without compromising meat yield or sensory acceptance [51]. Similar enrichment patterns were observed in Cobb 500 broilers fed 4% flaxseed meal, where breast and thigh ALA deposition increased and the PUFA:SFA ratio improved by 25–40% [33]. However, studies that used only 2% linseed, botanical mixtures with inherently high n-6:n-3 ratios, or short feeding durations below 28 days generally recorded modest or negligible incorporation of n-3 PUFA [83]. These contrasting outcomes explain the importance of inclusion level, bioavailability, and feeding phase in determining tissue deposition efficiency. Across sources, long-chain omega-3 fatty acids demonstrate the most direct impact. As it integrates easily into muscle phospholipids, lowers the n-6:n-3 ratio, and improves overall PUFA:SFA balance [82]. In contrast, ALA-rich ingredients require metabolic conversion before contributing to long-chain PUFA enrichment. This often results in smaller shifts in muscle fatty-acid profiles unless inclusion levels are high or feeding is prolonged. The addition of natural antioxidants such as turmeric, rosemary, and blackcurrant extracts, further stabilizes PUFA-rich tissues by reducing TBARS formation and preventing oxidative discoloration (Table 5). This enables meat or carcass to retain desirable sensory properties during chilled or frozen storage. Omega-3 fatty acid combined effects combined effects produce healthier lipid profiles and improve meat stability, meeting consumer demand for high-quality functional poultry products (Table 5). Studies show that 1–2% microalgal DHA or 2–4% fish or linseed oil administered during the finisher phase, producing DHA concentrations that meet or exceed functional food labelling thresholds (≥80–100 mg·100 g^−1^ meat). Future research should standardize analytical endpoints of sensory evaluation to better quantify the relationship between dietary inclusion rate, tissue deposition efficiency, and consumer-perceived quality.

### 3.7. Gut Morphology, Nutrient Digestibility, and Metabolic Responses

Dietary omega-3 sources consistently show positive effects on gut structure and nutrient assimilation when supplemented at adequate levels [87]. In Cobb 500 and Ross 308 broilers, inclusion of 4% flaxseed meal significantly increased jejunal villus height and villus-to-crypt ratio. [33]. The same study reported higher activities of α-amylase and invertase, confirming improvements in carbohydrate digestion, alongside shifts toward beneficial *Lactobacillus* spp. [33]. Comparable enhancements in villus morphology and mucosal stability were observed with a 30% extruded linseed-pea mixture, with or without *Lactobacillus acidophilus* co-supplementation [56]. This suggests that probiotics can amplify omega-3-mediated improvements in gut morphology [56]. Replacement of palm oil with 5% rice bran oil further improved intestinal fat absorption and elevated apparent fat digestibility [51]. Under high stocking density, 0.5% salmon oil supplementation enhanced apparent metabolizable energy, crude protein digestibility, and lipid absorption in Ross 308 broilers, while concurrently lowering serum cholesterol and triglycerides (Table 6). Although many studies show marked improvements, some report minimal or mixed effects under specific conditions (Table 6). For instance, 2% sacha inchi oil combined with medicinal plants did not significantly affect villus height or crypt depth [50]. Similarly, studies using low omega-3 inclusion levels (<2%), diets with high intrinsic n-6:n-3 ratios and short feeding durations (<28 days) typically observed limited deposition of omega-3 and negligible changes in gut morphology or digestibility indices [8,88]. Feeding trials with less than 1% microalgae, such as *Chlorella vulgaris*, showed stable villus structure and microbial profiles [15]. This further validates that low-dose supplementation may be insufficient to elicit structural or metabolic changes. These findings underscore that omega-3 efficacy is strongly influenced by inclusion level, oil bioavailability, and the duration of dietary exposure.

Broader microbiome responses also show dose-dependent effects. Diets containing flaxseed meal or extruded linseed–pea mixtures consistently increased lactic acid bacteria, reduced luminal ammonia, lowered intestinal pH, and improved mucosal enzyme activity [33,56]. Studies incorporating probiotics or herbal antioxidants, including rosemary and turmeric, reported additive benefits in maintaining microbial balance and suppressing oxidative gut inflammation [58,59]. In contrast, Cong et al. [50] found that 2% *sacha inchi* oil with medicinal plants did not significantly affect villus height or crypt depth. Mechanistic evidence across species helps contextualize these findings. Omega-3 incorporation into mucosal phospholipids increases membrane flexibility, villus height, and absorptive capacity [89], while also improving lipid transport and oxidation efficiency through transcriptional regulation of lipid metabolic enzymes and downregulation of cholesterol synthesis pathways [90]. Cross-species research further supports conserved ω-3–microbiome interactions. Human cohorts show enrichment of butyrate-producing genera and reduced pro-inflammatory taxa with higher ω-3 intake [91]. Whereas transgenic FAT-1 mice with a lowered n-6:n-3 ratio promote *Bifidobacteriaceae* abundance and suppress endotoxemia-related pathways [92]. Emerging metabolomic data reveal that omega-3 supplements modulate SCFAs, indoles, and lysophospholipids, which regulate tight-junction expression and mucosal integrity [93]. Likewise, *Lactobacillus–Bacillus* co-supplementation increases occludin, ZO-1, and claudin-1 expression, which improves the mucus layer. This establishes the central role of microbiota–metabolite interactions in epithelial resilience [94]. Future research should adopt harmonized metagenomic, transcriptomic, and metabolomic frameworks to elucidate host–microbiome–lipid interactions. Also, standardized histomorphometric and digestibility assays are needed to refine dose–response relationships and optimize inclusion strategies across production phases.

**Table 6:** Effects of omega-3 supplementation on Gut Morphology, Nutrient Digestibility, and Metabolic Responses.

References	Broiler Strain	Sample Size (Birds)	Duration (Days)	Omega-3 Source	Inclusion Level	Key Findings
[56]	Cobb 500	100	28	Flaxseed meal	4%	↑ Villus height; ↑ digestive enzymes; ↑ Lactobacilli; ↓ pathogenic bacteria.
[56]	Ross 308	480	42	Extruded linseed + pea ± probiotic	30%	↑ PUFA; ↓ SFA; ↑ cholesterol metabolism; ↑ gut microbial balance.
[95]	Ross 708 (breeders)	588	154	Microalgae vs. flax–pulse mixture	1–2.5%	Microalgae (DHA) ↑ villus height & crypt depth; ↑ nutrient absorption surface.
[28]	Ross 308	420	40	Salmon oil blend (Persia Fat)	0.50%	↑ Nutrient digestibility; ↑ gut integrity under stress.
[90]	Arbor Acres	192	42	Myristic acid (FA analog)	0.04%	↑ Lipid-transport gene expression; ↓ cholesterol synthesis; improved metabolic efficiency.
[96]	Ross 308	48	14–24	Fish oil (EPA + DHA)	Oral dosing	↓ IL-6 & triglycerides; ↑ HSP-70; improved heat tolerance and metabolic resilience.

↑: increase, ↓: decrease, AME = Apparent Metabolizable Energy; PUFA = Polyunsaturated Fatty Acid; SFA = Saturated Fatty Acid; HSP-70 = Heat Shock Protein 70; NPY = Neuropeptide Y; IL-6 = Interleukin-6; TG = Triglycerides.

## 4. Conclusions

This review synthesizing 69 studies (2020–2025) shows that omega-3 fatty acids consistently enhance broiler immunity, antioxidant capacity, gut functionality, and meat nutritional quality, with the strongest effects observed for long-chain sources such as fish oil and microalgal DHA. Plant-based ALA sources improved meat fatty-acid profiles and microbial balance but produced more variable performance outcomes due to limited conversion to EPA and DHA. These benefits remain dependent on inclusion level, dietary stability, antioxidant protection, and production conditions. Future research should apply integrated omics, microbial-metabolite profiling, and standardized dose-response trials to identify optimal inclusion strategies for precision nutrition and sustainable poultry production.

### Future Consideration

Omega-3 supplementation is now established as an effective strategy for improving poultry health and product quality, yet several research gaps remain. Future studies should employ standardized dose-response models across broiler genotypes and production phases to determine the minimal effective inclusion levels needed to achieve consistent physiological and enrichment outcomes. Advances in feed technology, including microencapsulated or antioxidant-fortified omega-3 ingredients, are essential for reducing peroxidation during storage and pelleting, thereby improving oxidative stability and tissue deposition efficiency. Multi-omics approaches such as integrating metabolomics, transcriptomics, and microbiome profiling are needed to clarify the mechanistic pathways through which omega-3s influence lipid metabolism, immune function, and gut integrity. Sustainability-oriented innovations such as valorizing oilseed by-products, incorporating algal co-residues, and adopting circular bioeconomy frameworks will further enhance the economic and environmental feasibility of omega-3 enrichment. Parallel research on consumer perception, labeling standards, and regulatory requirements will also be critical for enabling market adoption of omega-3 enriched poultry products. Overall, omega-3 fatty acid supplementation represents a robust and scalable strategy for strengthening the efficiency, resilience, and sustainability of poultry production. With optimized formulation, improved antioxidant protection, and circular feed innovations, the sector can deliver healthier, high-value products while advancing global goals for nutritional security and climate-smart agriculture.

## Figures and Tables

**Table 1:** Search engines for the study.

Database	Search Field/Operators Used	Search String	Filters and Limits Applied
Web of Science (Core Collection)	Topic (TS)—searches title, abstract, author keywords	TS = ((“omega-3 fatty acids” OR “n-3 polyunsaturated fatty acids” OR “EPA” OR “DHA” OR “alpha-linolenic acid” OR “fish oil” OR “flaxseed oil” OR “linseed oil” OR “microalgae” OR “algal oil”) AND (“broiler*” OR “chicken*” OR “poultry”) AND (“growth performance” OR “immune response” OR “health status” OR “oxidative stability” OR “fatty acid composition” OR “carcass quality” OR “meat quality”))	Years: 2020–2025; Language: English; Document type: Article
Scopus	TITLE-ABS-KEY (Title, Abstract, Keywords)	TITLE-ABS-KEY ((“omega-3” OR “n-3 PUFAs” OR “polyunsaturated fatty acids” OR “fish oil” OR “flaxseed oil” OR “linseed oil” OR “microalgae” OR “algal oil” OR “alpha-linolenic acid”) AND (“broiler*” OR “chicken*” OR “poultry”) AND (“growth performance” OR “immune response” OR “health status” OR “meat quality” OR “oxidative stability” OR “fatty acid composition” OR “carcass traits”))	Years: 2020–2025; Language: English; Document type: Article

**Table 3:** Effect of Omega-3 on Growth Performance and Feed Efficiency in Broiler Chicken.

References	Broiler Strain	Sample Size (Birds)	Duration (days)	Omega-3 Source	Inclusion Level	Key Findings
[63]	Ross 308	1600 (20 birds × 20 pens × 4 treatments)	35	Purified fish oil	0%, 0.05%, 0.10%, 0.15%	↑ body weight (higher BW at Day 21, ↑ body weight gain across all phases (Days 7–21, 22–35, 7–35).
[64]	Ross 308	420	40	Salmon oil blend (Persia Fat)	0.057% vs. 0.5%	0.5% ω-3 ↑ AME, ↑ digestibility, and ↑ BWG under stress.
[26]	Ross 308	420	42	Fish + linseed oil	1.5–4% (n-6:n-3 = 1.5–4.1)	Narrower n-6:n-3 ratios ↑ BWG and ↑ FCR; ↑ EPA/DHA in meat.
[22]	Cobb 500	600	42	ω-3 (0.25–1%) ± glutamine	0.25–1%	ω-3 + glutamine ↑ BWG by 25%; ↑ FCR efficiency.
[65]	Not specified	240	36	*Pulicaria jaubertii* powder	0–9 g/kg	3 g/kg ↑ BWG and ↑ FCR; ↓ ω-6:ω-3.
[66]	Ross 308 (male)	576	42	*Pulicaria gnaphalodes* powder (PGP)	0.1%, 0.2%, 0.3%	0.3% PGP ↑ BWG and ↓ FCR (grower, finisher & overall); performance comparable to AGP; 0.1% ineffective.
[57]	Hubbard	144	16–35	Omega-3 feed additive	5 & 10 kg/ton	↑ Growth rate and ↑ feed efficiency.
[51]	Ross 308	108	38	Rice bran oil (vs. palm oil)	5%	RBO ↑ ALA & ω-3; improved FCR; ↓ n-6:ω-3 ratio.
[48]	Ross 308	180	42	Nano-encapsulated flax oil	1 mL/kg BW	↑ BW, ↓ FCR; ↑ EPA/DHA.
[50]	Ho × Luong Phuong	288	70	Sacha inchi oil ± herbs	2% ± 1%	No effect on BWG or FCR

↑: increase, ↓: decrease, BWG = Body Weight Gain; FCR = Feed Conversion Ratio; AME = Apparent Metabolizable Energy; GPx = Glutathione Peroxidase; SOD = Superoxide Dismutase; MDA = Malondialdehyde; PUFA = Polyunsaturated Fatty Acid; ALA = α-Linolenic Acid.

**Table 4:** Effect of Omega-3 on Broiler’s Immune and Antioxidant Responses.

References	Broiler Strain	Sample Size	Duration (Days)	Omega-3 Source/Additive	Inclusion Level	Key Immune & Antioxidant Outcomes
[78]	Ross 308	340	42	Linseed, Echium oil, Fish oil, Algal biomass	15–50 g/kg	Linseed & algae ↑ NK cell activity; fish oil ↓ immune indices (dose-dependent).
[60]	Cobb 500	255	35	Fish oil vs. flaxseed oil	1.9–5%	Flax oil ↑ cytotoxic cell activity and ↑ n-3 PUFA in immune tissues.
[22]	Cobb 500	600	42	ω-3 (0.25–1%) ± glutamine	0.25–1%	ω-3 + glutamine ↑ GPx & SOD, ↓ MDA, and ↓ Eimeria lesions.
[79]	Broilers	80	42	CHI, Omega-3, CHI+Omega-3	G2: CHI 100 mg/kg; G3: Ω-3 0.2 mg/kg; G4: Ω-3 0.2 mg/kg on CHI	↑ WBC, ↑ lymphocytes, ↓ H:L ratio; ↑ serum proteins & immunoglobulins; ↑ ND & AI antibody titers; ↑ bursa, spleen & thymus indices; ↑ SOD & ↑ GSH
[80]	Ross 308	160	42	*Spinacia oleracea* extract ± Vitamin E	50 mg/kg	↑ SOD, ↑ CAT, ↓ MDA; ↑ n-3 PUFA deposition in meat.
[59]	Ross 308	350	42	Turmeric powder	0–10 g/kg	4 g/kg increased DHA & ω-3 in tissues; ↓ TBARS; ↑ oxidative stability.
[17]	Hubbard Flex	120	35	Rosemary + blackcurrant extracts	2.5–5 g/kg	↓ MDA in frozen meat; ↑ total antioxidant capacity.
[81]	Ross 308	240	42	Flax oil + Se + Vitamin E	1.5% + 0.3 mg Se + 200 IU Vit E	↑ SOD, CAT, GPx, ↓ MDA; strong antioxidant synergy.
[80]	Broilers (Trial 2)	100	18	*Spinacia oleracea* extract	50 mg/kg	↑ SOD, CAT, GPx, ↓ NO, ↓ lesion scores; ↑ goblet cell density.

↑: increase, ↓: decrease, SOD = Superoxide Dismutase; CAT = Catalase; GPx = Glutathione Peroxidase; MDA = Malondialdehyde; DHA = Docosahexaenoic Acid; EPA = Eicosapentaenoic Acid; Vit E = Vitamin E; GSH = Reduced Glutathione.

**Table 5:** Effect of Omega-3 on Broiler’s Meat Quality and Fatty Acid Composition.

Reference	Broiler Strain	Sample Size	Duration (Days)	Omega-3 Source	Inclusion Level	Key Findings
[54]	Ross 308	_	21–42	*Aurantiochytrium limacinum* (microalgae)	0–2%	↑ DHA (97–156 mg/100 g meat); no negative effect on growth.
[67]	Ross 308	240	35	Algal oil ± Vitamin E	1.5% + 200 IU VE	3× ↑ DHA, ↑ SOD & CAT, ↓ MDA/TBARS; improved oxidative stability.
[81]	Ross 708	144	18–35	Flaxseed vs. fish oil	50 g/kg	Fish oil ↑ DHA; flax
[37]	Cobb 500	55	35	Ahiflower (SDA) vs. flaxseed	7.5–22.5 g/kg	SDA ↑ EPA/DHA conversion; 7.5 g/kg most efficient for n-3 enrichment.
[84]	Ross 308	240	42	Walnut meal + cranberry leaves	6% + 1–2%	↑ Long-chain n-3 PUFA; ↓ oxidative stress; ↑ meat oxidative stability.
[85]	Ross 308	200	42	Chickpea replacing soybean meal	50% protein replacement	↑ PUFA; ↓ Atherogenic (AI) & Thrombogenic (TI) indices.
[53]	Slow-growing Korat	700	42 (3–9 weeks)	Tuna oil replacing rice bran oil	1.5–4.5%	↑ EPA, ↑ DHA, ↑ total n-3 PUFA; ↓ n-6 PUFA & ↓ n-6:n-3 ratio; no change in growth or meat quality.
[23]	Ross 308	270	42	*Camelina sativa* meal (solvent extracted)	0–25%	High inclusion (≥10%) ↓ growth, FCR & carcass yield; not suitable for broilers due to anti-nutritional effects.
[58]	Ross 308	96	35	Flax oil ± quercetin	50% ω-3 source	↑ ALA deposition; ↓ MDA; ↑ oxidative stability in meat.
[86]	Arbor Acres	540	56	*Prosopis africana* oil	0–800 mg/kg	↑ PUFA, ↓ SFA; improved flavour and consumer acceptability.

↑: increase, ↓: decrease, DHA = Docosahexaenoic Acid; ALA = α-Linolenic Acid; MDA = Malondialdehyde; TAC = Total Antioxidant Capacity; LC n-3 PUFA = Long-chain Omega-3 Polyunsaturated Fatty Acid.

## Data Availability

The original contributions presented in this study are included in the article. Further inquiries can be directed at the corresponding author.

## Abbreviations

The following abbreviations are used in this manuscript:

ALA	Alpha-Linolenic Acid
AI	Atherogenic Index
AME	Apparent Metabolizable Energy
BW	Body Weight
BWG	Body Weight Gain
CAT	Catalase
DHA	Docosahexaenoic Acid
EPA	Eicosapentaenoic Acid
ET-1	Endothelin-1
FA	Fatty Acid
FADS1	Fatty Acid Desaturase 1
FADS2	Fatty Acid Desaturase 2
FCR	Feed Conversion Ratio
GSH	Reduced Glutathione
GPx	Glutathione Peroxidase
H/L ratio	Heterophil-to-Lymphocyte Ratio
HSP-70	Heat Shock Protein 70
IgA/IgG	Immunoglobulin A/Immunoglobulin G
IL-6	Interleukin-6
LC-PUFA	Long Chain Polyunsaturated Fatty Acid
L-FABP	Liver Fatty Acid Binding Protein
MDA	Malondialdehyde
NO	Nitric Oxide
NPY	Neuropeptide Y
PUFA	Polyunsaturated Fatty Acid
PPARα	Peroxisome Proliferator-Activated Receptor Alpha
RCB	Randomized Complete Block (design)
RBO	Rice Bran Oil
SFA	Saturated Fatty Acid
SOD	Superoxide Dismutase
TBARS	Thiobarbituric Acid Reactive Substances
TG	Triglycerides
TI	Thrombogenic Index
Vit E/VE	Vitamin E
ω-3/n-3	Omega-3 Fatty Acids
